# HBV Genotypic Variability in Cuba

**DOI:** 10.1371/journal.pone.0118959

**Published:** 2015-03-05

**Authors:** Carmen L. Loureiro, Julio C. Aguilar, Jorge Aguiar, Verena Muzio, Eduardo Pentón, Daymir Garcia, Gerardo Guillen, Flor H. Pujol

**Affiliations:** 1 Laboratorio de Virología Molecular, Centro de Microbiología y Biología Ceular, Instituto Venezolano de Investigaciones Científicas, Caracas, Venezuela; 2 Centro de Ingeniería Genética y Biotecnología, La Habana, Cuba; CRCL-INSERM, FRANCE

## Abstract

The genetic diversity of HBV in human population is often a reflection of its genetic admixture. The aim of this study was to explore the genotypic diversity of HBV in Cuba. The S genomic region of Cuban HBV isolates was sequenced and for selected isolates the complete genome or precore-core sequence was analyzed. The most frequent genotype was A (167/250, 67%), mainly A2 (149, 60%) but also A1 and one A4. A total of 77 isolates were classified as genotype D (31%), with co-circulation of several subgenotypes (56 D4, 2 D1, 5 D2, 7 D3/6 and 7 D7). Three isolates belonged to genotype E, two to H and one to B3. Complete genome sequence analysis of selected isolates confirmed the phylogenetic analysis performed with the S region. Mutations or polymorphisms in precore region were more common among genotype D compared to genotype A isolates. The HBV genotypic distribution in this Caribbean island correlates with the Y lineage genetic background of the population, where a European and African origin prevails. HBV genotypes E, B3 and H isolates might represent more recent introductions.

## Introduction

Even though the highest HBV prevalence is found in the Eastern Southern Asia and in the Southern and Equatorial Africa, around 13 million persons are infected with HBV in America and around 11 million of them are found in Latin America, a region exhibiting an intermediate prevalence of infection, varying from 2 to 7% [1]. HBV has been classified in 8 genotypes (A–H), exhibiting a minimum divergence of 8% in the complete genome sequences [2]. In addition, 2 new genotypes, I and J, have been proposed [3]. Genotypes A and D are also widely distributed in all the continents. Genotypes B and C are found mainly in South East Asia and the Far East, while genotype E circulates in sub-Saharan West Africa [4]. Several lines of evidence suggest that HBV genotype E might be a recent genotype: the low intragenotypic variation exhibited by these strains and the fact that it was not introduced to the Americas during slave trade, suggesting a posterior origin [5,6]. Genotype G has been reported in the US, Mexico and Europe, but its distribution is not fully known [7]. HBV genotype F is the most divergent of the HBV genotypes, autochthonous to South America and highly predominant in the region [8–10]. HBV genotype H is closely related to genotype F and is prevalent Central and North America [11,12].

Although HBV genotypes F and H are indigenous to America, their prevalence varies markedly among different countries. In the Southern region of South America, HBV genotype A prevails over genotype F, while in the Northern region genotype F is highly predominant. In Brazil, the genotype distribution is related with the immigration pattern, and a high prevalence of genotype A can be seen in African-Brazilians. HBV genotype H has only been found in Central America and is found circulating either with genotype A or F. The relative frequency of HBV genotype F in Latin America is in close correlation with the degree of admixture of the general population with Amerindians, in many cases through the maternal contribution to the genetic pool. For example, in Colombia and Venezuela, where the frequency of HBV genotype F is around 80% in the general population [13], the majority of mtDNA is of Amerindian origin [14,15]. In contrast, in Brazil, the Amerindian contribution to the mtDNA genetic pool is only 33%, with a more significant contribution of African mtDNA [16]. HBV genotype A is more common than HBV genotype F in this country [17,18].

Cuba exhibits an intermediate prevalence of HBV, which has been declining with the active vaccination program in the country. The prevalence of HBV surface antigen (HBsAg) has been reducing in blood donors from more than 1% before the starting of vaccination in 1992, to around 0.5% in the last years [19]. No information is available on the genotypic diversity of HBV in the island. The aim of this study was to explore the genotypic diversity of HBV in Cuba.

## Materials and Methods

This study was approved by Comité de Revisión y Etica of Sanctis Spiritus, Cuba and by Comité de Bioética del IVIC, Venezuela. The S (700 nt and 1200 nt respectively), X gene and precore-core, and complete genomic regions were amplified from 250, 14, 109 and 19 HBsAg positive sera, respectively, as previously described [13,20,21]. These sera were from Cuban untreated patients, collected in 2006 (samples Cuba) and 2013 (samples Cuba “a”), obtained with written informed consent of the donor. PCR purified fragments were sent to Macrogen Sequencing Service (Macrogen, Korea) for sequencing. Both strands of DNA were sequenced. Sequence alignment and phylogenetic analysis by the Neighbor Joining method (500 bootstrap replicas) with genetic distances evaluated with Kimura 2 parameters corrections, were conducted using DNAman 5.2.2 (Lynnon Bio Soft, Canada). Reference sequences from the different genotypes and subgenotypes were included in the phylogenetic analysis, as well as sequences closely related to the Cuban sequences analyzed, obtained by BLAST analysis. Electropherotypes were also visually inspected to detect the presence of variants in specific nucleotides, associated to stop codons or other relevant mutations or polymorphisms. Nucleotide sequence data have been deposited into the GenBank database under the accession numbers KM606642-KM606972.

Statistical differences were evaluated by the Chi-Squares test with Yates correction, or Fisher Exact test (when a cell number under 5), according to a computerized Epi Info program, version 3.5.3 (Centers for Disease Control and Prevention, Atlanta, GA).

## Results

**Fig 1:**
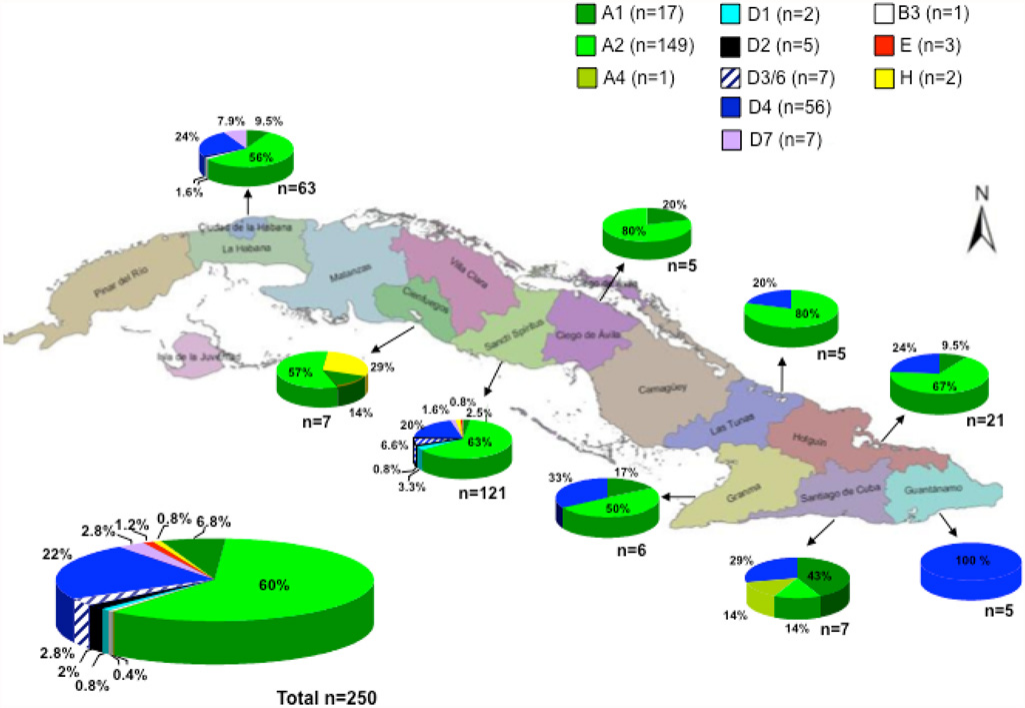
HBV genotype distribution in Cuba.

A total of 250 sera from HBV infected patients were analyzed. Most of the patients were chronic carriers and none of them presented with hepatocellular carcinoma (HCC). Fig. 1 shows the prevalence of HBV genotypes in these Cuban patients, according to phylogenetic analysis of a partial S genomic region (700 nt). A high genetic diversity was found among Cuban isolates. The most frequent genotype was A (167/250, 67%), mainly A2 (149, 60%) but also A1 and one A4. A total of 77 isolates were classified as genotype D (31%), with co-circulation of several subgenotypes (56 D4, 2 D1, 5 D2, 7 D3/6 and 7 D7). Three isolates belonged to genotype E, two to genotype H, and one to genotype B3. For the B3 isolate, only 700 nt from the S region were available for phylogenetic analysis. This isolate was closely related to an isolate form Australia, but also with isolates from USA and Vietnam (data not shown). No significant difference was found in HBV genotype distribution among the studied Cuban localities (Fig. 1). In addition, when examining the sequence electropherotypes, evidence suggesting mixed A/D infection was found in 9 isolates (data not shown). In addition, for 2 isolates, genotype deduced from the core region was different from the one assigned by analysis of the S region: Cuba 15a (genotype A2 in the S region and A1 in the core region), and Cuba99a (genotype D4 in the S region and A2 in the core one), suggesting mixed infection or recombination.

**Fig 2:**
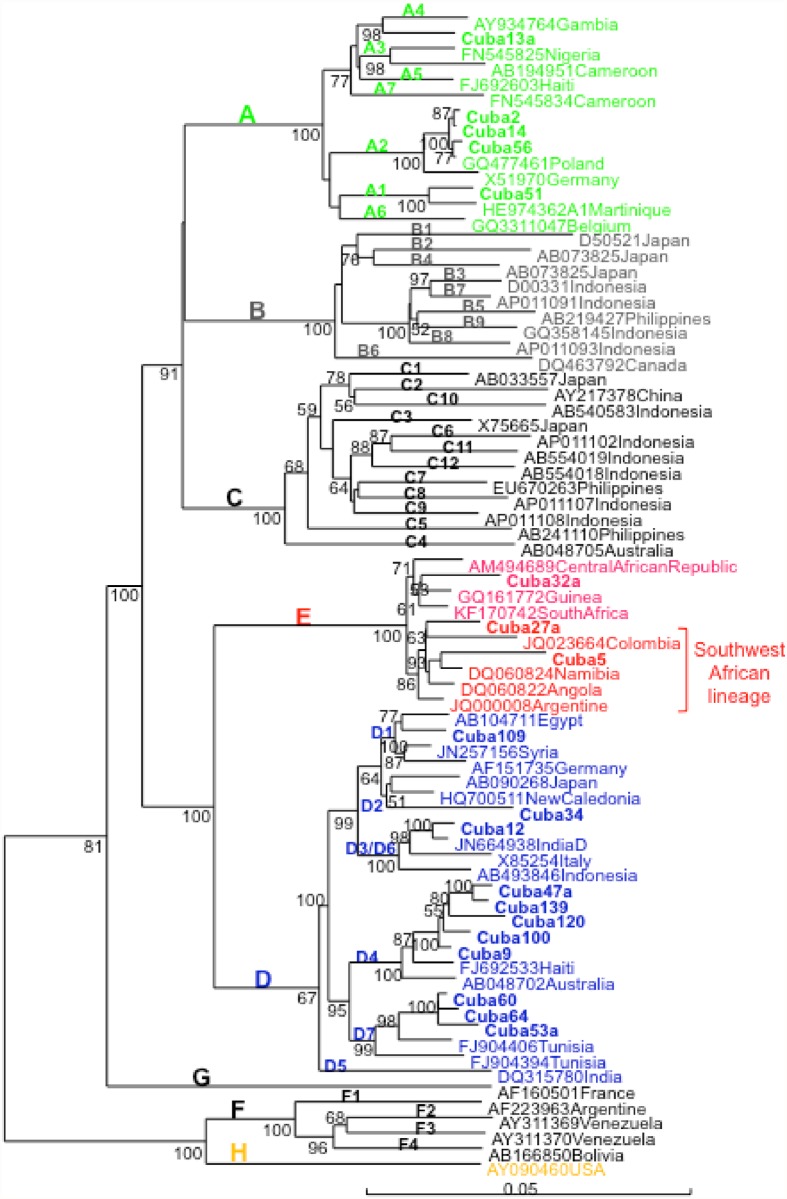
Phylogenetic analysis of HBV complete genome of Cuban isolates. Genetic distance was estimated by Kimura 2 parameters and phylogenetic tree was constructed with the Neighbor joining method. Numbers at each node correspond to bootstrap values (greater than 50%) obtained with 500 replicates. Isolates are designated by their GenBank accession number, followed by their country of origin, except for Cuban ones which are shown in bold.

Complete genome sequence analysis of 19 selected isolates confirmed the phylogenetic analysis performed with the S region and allowed analyzing phylogenetic relatedness of Cuban HBV isolates with the ones from other countries (Fig. 2). BLAST analysis was performed using the Cuban sequences and the most similar sequence available at GenBank was included in the phylogenetic tree. Cuban HBV A1 isolates were closely related to an isolate from Martinique, A2 isolates to a European isolate (Poland), and A4 with an African (Gambian) isolate. A2 isolates were also related to Spanish isolates (GenBank accession numbers AJ627226 and AJ627228, data not shown). D1 isolate was closely related to an isolate from Egypt, D2 with Asian isolates. D3/D6 isolate–D3 and D6 subgenotypes were recently regrouped in a single subgenotype [22]—was closely related to an Indian isolate, D4 isolates were closely related to HBV an isolate from Haiti and D7 isolates were closely related to Tunisian isolates, where this subgenotype was first identified [23]. D4 and D7 Cuban isolates formed monophyletic clusters inside this subgenotype. Cuban D4 isolates were also closely related to a Spanish D4 isolate (Accession number AJ627219) and more distantly related to D4 isolates from Maranhao Brazil (Accession number KJ470898) (data not shown). From the 3 genotype E isolates, two were more closely related to the HBV genotype E isolate were from Argentine, Colombia, Angola, Namibia and Congo, the recently proposed South West African lineage [24]. However, a third Cuban genotype E isolate grouped outside this lineage.

**Fig 3:**
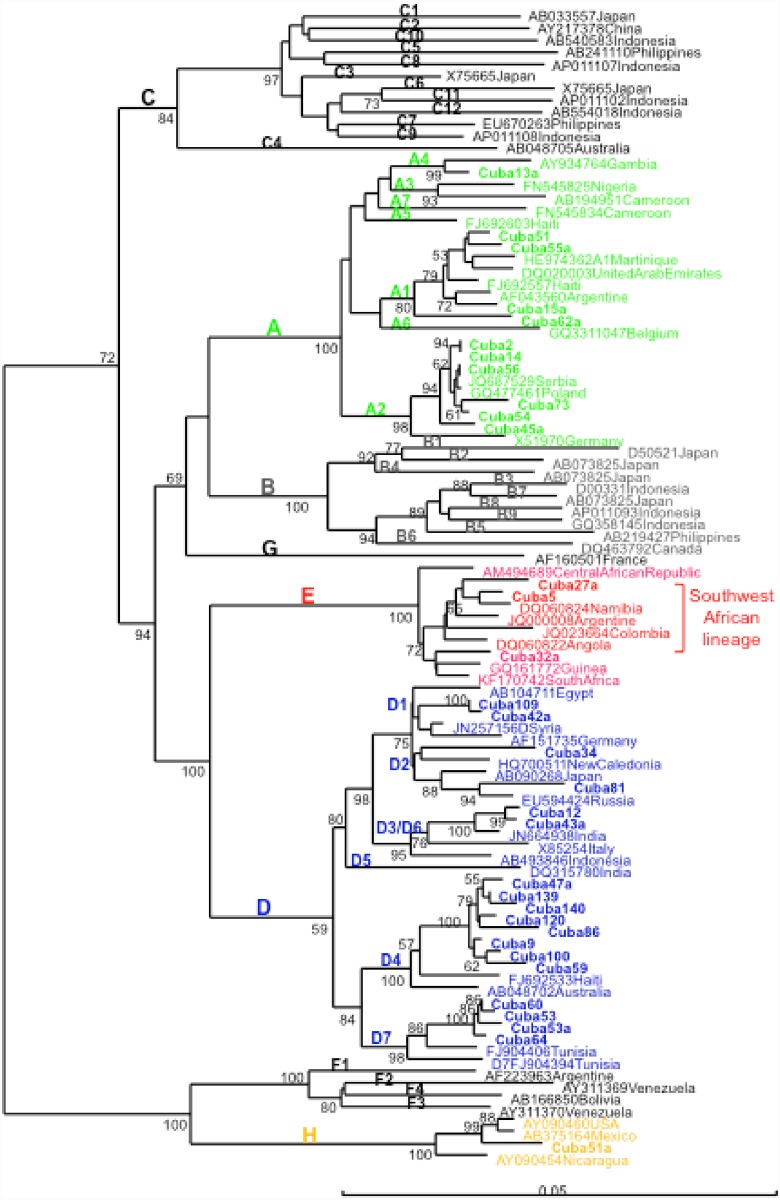
Phylogenetic analysis of HBV S region (1245 nt) of Cuban isolates. Genetic distance was estimated by Kimura 2 parameters and phylogenetic tree was constructed with the Neighbor joining method. Numbers at each node correspond to bootstrap values (greater than 50%) obtained with 500 replicates. Isolates are designated by their GenBank accession number, followed by their country of origin, except for Cuban ones which are shown in bold.

In addition, genomic analysis of large S genomic region (more than 1100 nucleotides) allowed identifying closely related isolates in more samples for which complete genome sequence was not available, with high bootstrap support (Fig. 3). The same topology was found compared to the complete genome analysis, supporting the adequacy of this region to analyze genetic relatedness among strains. Two D3/D6 isolates formed a closely related cluster, related also to an Indian isolate, as described previously. D2 isolates did not form a monophyletic cluster, being one isolate related to a New Caledonian one and another to a Russian isolate. One HBV genotype H isolate was more closely related to isolates from Mexico and USA than from Nicaragua.

Subtype and genotype distribution of Cuban isolates were highly correlated (Table 1). All but one genotype A isolates were subtype adw2, as expected. All but 12 of the genotype D isolates were subtype ayw4, being only 3 subgenotype D4 isolates subtype ayw2, which is a more common subtype found in genotype D isolates [2]. The 3 genotype E isolates were subtype ayw4 and the two genotype H ones subtype adw4, as expected.

**Table 1:** Genotype and subtype in Cuban HBV isolates.

Subtype	adw2	adw4	ayw1	ayw2	ayw3	ayw4
Genotype						
**A1**	17					
**A2**	148		1			
**A4**			1			
**B3**			1			
**D1**						2
**D2**					1	4
**D3/D6**					5	2
**D4**				4		52
**D7**						7
**E**						3
**H**		2				
**Total**	165	2	3	4	6	70

**Fig 4:**
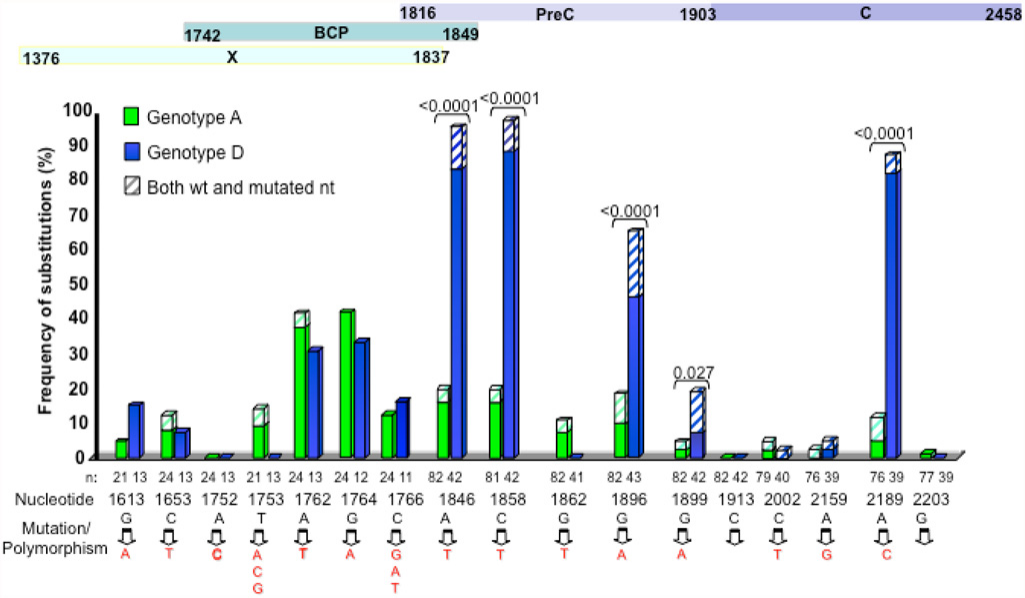
Frequency of X, BCP, precore and core mutations/polymorphisms in genotype A and D Cuban isolates. Nucleotide position and mutation is shown. Hatched part of the bar reflects the frequency of mixed wild type and mutated sequence. Significant differences in frequency between the two genotypes are shown with a bracket.

The presence of mutations or polymorphisms in basal core promoter (BCP) and precore (PC) regions were analyzed and compared between the two more frequent genotypes, A and D (Fig. 4). A tendency toward a higher frequency in HBV genotype A isolates to harbor the BCP mutations A1762T/G1764T was found, although not statistically significant. Some polymorphisms were more frequently found in genotype D compared to genotype A: C1858T, predisposing to the precore mutation G1896A, which was also found significantly more frequently in genotype D isolates. The other precore mutation G1899A was also found significantly more frequently in genotype D isolates, although this mutation was less frequent that the G1896A one. Two other polymorphisms, A1846T and A2189C, were found at high frequency in genotype D isolates, and were almost absent in genotype A ones.

In addition, deletions were found in the core region in 3 isolates, leading to truncated core proteins (Table 2). Another deletion leading to a C-terminal truncated X protein was found in one D2 isolate. Three stop codon mutations were relatively frequent in the Surface protein: C69*, W182* and L216*, circulating mostly as variants along with the wild type codon (Table 2). The combination C69*W182* was frequently found and these mutations were significantly more frequent in the D4 isolates compared to the A2 ones. Finally, 4 isolates harbored mutations in the Polymerase protein, associated to resistance to Lamivudine.

**Table 2:** Mutations in precore, core, surface and polymerase of Cuban HBV isolates.

Region	N isolates	Mutation	Subgenotype (n/tot)^1^
**Surface**	250	C69*	A2(**4**/149)D4^3^(**12**/56), p<0.001
		W182*	A2(**12**/149)D4^3^ (**12**/56), p = 0.01
			D2(**1**/5)D3(**1**/7)
		L216*	A1(1/17)A2^4^(7/149)
			D2(1/5)D4(1/56)E(2/3)
**Polymerase**	250	YIDD	A2(2/149)
		YVDD	A2(2/149)
**Precore** ^**2**^	125	Stop cod 2	A2(**1**)
		OF del cod 9	D3/D6(1)
		OF ins cod 8	A2(1)
**Core** ^**2**^	119	OF del cod 63–76	D4(2)
		Stop cod 92 and del	D4(1)

^1^Mutations found circulating with wild type variant are shown in bold.

^2^Mutations other than the ones analyzed in Fig. 4.

^3^One D4 isolate exhibits both mutation without wild type variant.

^4^One D4, one E and 3 A2 isolates exhibit L216

* mutation without wild type variant.

OF: Out of frame. Cod: codon. Del: deletion. Ins: insertion

## Discussion

The genetic diversity of HBV in human population is often a reflection of its genetic admixture and is also influenced by more recent human international migrations and relations [17,25,26]. The Native American first people in Cuba were almost replaced by European and slaves from Africa [27]. HLA haplotypes of European and African origin prevails, although some Amerindian HLA alleles are found [28]. More recent studies have shown that Native American maternal genes (mtDNA) are still prevalent in the island (33% in average), while they are not found in the male Y chromosome pool [27]. Then a strong sex bias is found between the paternal and maternal ancestries in Cuba. In the paternal ancestry, the European genetic contribution is almost 80%, with only 20% African one [27]. The most frequent European immigrants were Spanish from the Canary Islands, being some haplogroups characteristic of these Spanish islands found frequently in Cuba [27].

The HBV genotype distribution in Cuba is interestingly correlated with its phylogeographic structure of the Y-chromosome lineages [27]. The most common subgenotype was the European A2, followed by the African subgenotype D4. The A2 isolates were closely related to isolates from Eastern Europe (Figs. 2 and 3). They were also related to Spanish HBV A2 isolates. The Cuban A2 isolates did not form a monophyletic cluster (Fig. 3). Being the most common subgenotype, multiple introductions might have been expected, probably most from the first Spanish immigrants, but also arising from more recent contacts with the former Soviet Union and related countries. In Spain, the most common HBV genotype is D, followed by A [29,30]. Analysis of the only 14 complete genome HBV sequences available at GenBank allows suggesting that the most common subgenotype circulating in Spain is D2 (GenBank accession numbers AJ627215, AJ6272156, AJ627218, AJ627220, AJ627222, AJ627223), followed by A2 (GenBank accession numbers AJ627226-AJ627228). However, HBV D2 isolates are not common in Cuba, with only 2% prevalence (Fig. 1). Interestingly, both A2 and D2 Cuban isolates did not form monophyletic clusters, suggesting multiple introductions of these subgenotypes. This situation is in agreement with multiples introductions of HBV in the island by Spanish colonizers.

In contrast, the A1 isolates, closely related to isolates found in Haiti and Martinique (Fig. 3), might have been imported during the slave trade to the island, as in the other Caribbean islands [31,32]. In Haiti and Martinique, however, the A1 subgenotype is by far the most prevalent, situation not shared with Cuba. The higher prevalence of A1 in Haiti and Martinique is in agreement with a higher contribution of an African genetic background in these islands, compared to Cuba. The Cuban A1 isolates did not form a monophyletic cluster (Fig. 3), suggesting multiples introductions during slave trade. Kramvis and Paraskevis [26] suggested that phylogenetic analysis of HBV A1 isolates can be used to trace human migrations outside and from Africa. Indeed, the A1 Cuban isolates, although not forming a monophyletic group, were related to isolates from the Asian-American clade, which groups sequences from Haiti, Martinique, Brazil and other Latin American countries [26,33]. Another A4 isolate was related to an isolate from Gambia, suggesting again a probable African origin.

Likewise, the Cuban D4 isolates were closely related to an isolate from Haiti, suggesting a route of introduction for this subgenotype similar to the A1 one. This subgenotype is not frequent however in Haiti, or in Martnique [31,32]. HBV subgenotype D4 was also found very frequently in Maranhao state, Northeast Brazil, together with subgenotype A1 [34]. The authors suggest that HBV D4 might have been more prevalent in Africa in the past, during slave trade. The Cuban D4 isolates were not closely related with the Brazilian ones (data not shown). As for Maranhao´s isolates, Cuban ones formed a monophyletic cluster, suggesting single introduction of this subgenotype in the island, independent from the one occurred in the Brazilian region. The most frequent subtype associated to Cuban HBV D4 isolates was ayw4, while the isolates form Maranhao and Spain were subtype ayw2, as more usually found in this subgenotype. Altogether these results suggest an independent introduction of D4 subgenotype from Africa to Cuba.

Cuban D7 isolates were related with Tunisian isolates, but formed a monophyletic group. This subgenotype predominates also in Morocco [35]. No information is available about the genetic diversity of HBV in Libya, but this subgenotype might also circulate in this country, with which the island displayed some interaction in the past century. Cuban D3/D6 isolates were related to an Indian isolate (Fig. 3) and formed a monophyletic cluster (data not shown). A single introduction from this Asiatic country might be predicted from these results. Cuban D1 isolates were related to isolates from the Middle East and Egypt. The Cuban isolates did not form a monophyletic cluster (data not shown), suggesting multiple introductions from Middle East and Northern Africa.

None of the Cuban isolates belonged to the American genotype F, and only two genotype H isolates were found. This situation is in agreement with the absence of Amerindian genetic background at the phylogeographic structure of the Y-chromosome lineages found in the Cuban general population. The genotype H isolates found in Cuba might be related to the interaction of this island with Nicaragua in the past century. Alternatively, the presence of this American genotype might be related to the fact that Amerindian haplogroups are still found in maternal specific gene pool of the Cuban population. Indeed, one Cuban H isolate was more closely related to a Mexican isolate than to the Nicaraguan ones (Fig. 3). The origin of Native Americans in the Caribbean, such as Siboneys and Tainos, is a controversial issue. Two possible origins have been hypothesized for the origin of Native American Cuban people: coming from the Orinoco Valley, but also from Yucatan or Florida peninsulas [27]. The finding of HBV genotype H in Cuba, instead of genotype F, if related to the Native American genetic pool of the population, might favor the second hypothesis.

No HBV genotype G was also found among the Cuban isolates. Genotype G is not frequent in Latin America. In Venezuela, for example, only one isolate genotype G has been described, infecting an HIV-infected patient [36]. None of these Cuban patients were co-infected with HIV.

Three genotype E isolates were also found, probably due to recent introductions to the island. Bayesian coalescence studies suggest that HBV E is a recent genotype, no more than 130 years old, which did not exist during slave trade, explaining why it is not frequently found in Afrodescendent populations in the Americas [17,37]. Thus, the presence of this genotype in the island might not be related to the slave trade but instead be related to a more recent introduction, probably from Angola. Indeed, one of the patients infected with HBV genotype E was an Angolan citizen. HBV genotype E is highly predominant in Angola [38,39]. From November 1975 to 1991, Cuban military engagement occurred several times in Angola, involving more than 25,000 troops [40]. It is probable then that during this period HBV genotype E might have being introduced to the island. Two of the HBV E isolates were grouped in the South West African lineage, which comprises Angolan isolates, supporting this hypothesis. The other HBV E isolate was related to an isolate from Guinea. It is noteworthy to highlight at this point the medical missions sent by Cuba to many African countries too.

HBV displays genome variability, particularly at the precore/core and the BCP region, which have been associated to poor prognostic and HCC in infected patients [41]. These mutations or polymorphisms occur more frequently in some genotypes compared to others. Many mutations, polymorphisms and even deletions were found in the BCP, precore/core and X protein, particularly in HBV genotype D isolates, as expected. The frequency of these mutations/polymorphisms is similar to the one described previously in non-HCC bearing patients, in other locations [42]. Although found in non HCC bearing patients, the presence of a truncated form of the X protein in one patient, which has been associated to HCC [43], together with BCP mutations, warrant a careful follow up of these patients in order to prevent metastatic development. Mutations conferring resistance to lamivudine were found in 4 patients: two patients harbored the YIDD and two the YVDD one. Each pair of sequences were closely related at the S gene (data not shown), suggesting transmission of resistant viruses to Naïve patients. Three stop codon mutations were frequently found in the Surface protein, mostly circulating in the isolates as variants with the wild type amino acid. C69* mutation has been associated with occult HBV infection [44], and causes an impaired secretion of this antigen [45]. Moreover, this mutation is associated with S78T mutation in the Polymerase protein, which has been correlated with the use of Adefovir in treated patients [44]. W182* mutation has been associated to progression to liver disease and HCC [46] and occult infection [47]. L216* was found circulating in several genotypes A1, A2, D4 and E, and was found previously in one patient with occult infection [48].

In conclusion, a high HBV genetic diversity was found in Cuba, and a good correlation could be established between the genetic diversity, the genetic pool of the population and the history of human migrations and relations of the island.
